# Intestinal colonization against *Vibrio cholerae*: host and microbial resistance mechanisms

**DOI:** 10.3934/microbiol.2023019

**Published:** 2023-04-13

**Authors:** Abdullahi Yusuf Muhammad, Malik Amonov, Chandrika Murugaiah, Atif Amin Baig, Marina Yusoff

**Affiliations:** 1 Faculty of Medicine, Universiti Sultan Zainal Abidin, Malaysia; 2 Faculty of Medicine, Universiti Malaysia Sabah, Malaysia; 3 University Institute of Public Health, Faculty of Allied Health Sciences, The University of Lahore, Pakistan

**Keywords:** *Vibrio cholerae*, colonization resistance, cholera, enteric pathogen, cholera susceptibility

## Abstract

*Vibrio cholerae* is a non-invasive enteric pathogen known to cause a major public health problem called cholera. The pathogen inhabits the aquatic environment while outside the human host, it is transmitted into the host easily through ingesting contaminated food and water containing the vibrios, thus causing diarrhoea and vomiting. *V. cholerae* must resist several layers of colonization resistance mechanisms derived from the host or the gut commensals to successfully survive, grow, and colonize the distal intestinal epithelium, thus causing an infection. The colonization resistance mechanisms derived from the host are not specific to *V. cholerae* but to all invading pathogens. However, some of the gut commensal-derived colonization resistance may be more specific to the pathogen, making it more challenging to overcome. Consequently, the pathogen has evolved well-coordinated mechanisms that sense and utilize the anti-colonization factors to modulate events that promote its survival and colonization in the gut. This review is aimed at discussing how *V. cholerae* interacts and resists both host- and microbe-specific colonization resistance mechanisms to cause infection.

## 1. Introduction

Cholera is a gastrointestinal infection caused by an enteropathogenic gram-negative bacterium, *Vibrio cholerae*. The infection is characterized by voluminous watery stool with a characteristic rice-water colouration and painless vomiting resulting in hypovolemic shock and eventually death if left untreated [1]. Cholera has had seven pandemics since 1871, and the 7^th^ pandemic is still ongoing, affecting millions of people worldwide with an estimated average of more than 100,000 deaths yearly, as reported by World Health Organization (WHO) [2]. Outside its host, *V. cholerae* persistently inhabits the aquatic environment, including rivers and coastal waters, forming biofilms in association with marine organisms such as zooplankton and copepods [3], and can eventually spread to its host populations such as humans through contamination of food and water. Only 2 serogroups out of over 200 serogroups of *V. cholerae* isolated are known to be responsible for epidemic cholera infection, the O1, and O139 serogroups. The O139 serogroup is only isolated lately, but the O1 serogroup is more primitive and is known to be responsible for all the pandemics [4]. However, each serogroup can be classified into Classical and El Tor biotypes; while the El Tor biotype is known to be responsible for the ongoing cholera pandemic since 1961, the classical biotype is arguably known to be the causative agent of the previous six pandemics since 1871. In addition, it is more virulent and causes more severe diarrhoea than its El Tor counterpart [5]. Yet, the El Tor biotype is currently known to predominate the niches where *V. cholerae* O1 can be located and isolated; this may be due to the strain being shown to be more advanced and evolved in the aquatic environment, showing a better survival rate compared to the classical biotypes [6].

Once orally ingested, this salt-tolerant bacterium must survive the new hostile environment of the human gut, colonize the proximal and distal intestinal epithelium, and then produce cholera toxin responsible for the onset of cholera symptoms [7]. Once in the host, *V. cholerae* senses certain environmental signals that are central to regulating the expression of genes crucial for the survival, colonization, and pathogenicity of the microorganism in the human host. These environmental signals are from both the host and the gut commensal microbiotas that are in a symbiotic relationship with the host, protecting against pathogenic microbes through mechanisms such as the production of antimicrobial peptides, nutrient competition, and acidic pH [8]. Interestingly, *V. cholerae* uses these colonization resistance mechanisms to modulate its virulence, antagonizing them [9],[10]. To colonize the gut, *V. cholerae* must overcome the colonization resistance mechanisms by devising antagonistic means that will neutralize the effect of the resistance. Several factors are known to constitute *V. cholerae* virulence, but cholera toxin (CT) is the master virulence known to be responsible for the cholera symptom, diarrhoea. Thus, strains that are unable to produce CT are unable to cause cholera infection even after successful colonization in the gut. Toxin-coregulated pilus (TCP) and outer membrane proteins (OMPs) are known to play a vital role in the *V. cholerae* colonization and survival in the gut, and all three major virulence factors are known to be under tight regulation of *ToxR* regulon [11].

Most colonization resistance mechanisms are not specific to *V. cholerae*, but rather general to enteric pathogens or even gut commensals [12]. The gut microbiome plays a dual role and is central to overall colonization resistance, providing specific colonization resistance mechanisms such as bacteriocins and short-chain fatty acids (SCFA) and stimulating the host immune system in providing colonization resistance, such as the production of mucus. *V. cholerae* infection is primarily affected by the composition of an individual gut microbiota; a healthy gut is more resistant to *V. cholerae* infection than a dysbiotic gut from a malnourished or unhealthy individual. A dysbiotic gut is more susceptible to *V. cholerae* infection or other enteric infections due to weakened immunity due to a distorted microbiome. Many decades ago, this phenomenon was shown by Sack & Miller, who demonstrated that mice whose gut microbiota has been depleted by prior treatment with antibiotics are more susceptible to *V. cholerae* colonization compared with mice whose gut microbiota is unaffected [13]. Several studies have been conducted on the mechanisms that *V. cholerae* utilize to overcome colonization resistance and cause infection. This review is focused on summarizing the existing literature on how *V. cholerae* resists host and gut microbiota-specific colonization resistance mechanisms, how that affects its gene expression, and the role of gut microbiota in *V. cholerae* infection.

## 2. Host-specific colonization resistance mechanisms

The human gut harbours various microbes such as bacteria, protozoa, viruses, and fungi, some of which are commensals while others can inhabit as pathogens. Thus, the human body is equipped with numerous mechanisms to limit the growth, colonization, and abundance of invading pathogens while maintaining the gut commensals composition. The innate immune system is crucial and is responsible for coordinating and induction of signalling pathways that modulate mechanisms to recognize, capture, and incapacitate or destroy enteropathogens [14]. This response is less specific and serves as the first line of defence in protecting the body against pathogens since the adaptive immune response takes time to develop. The host-specific colonization resistance mechanisms are among the innate immune response and are discussed below regarding *V. cholerae* infection and summarized in Table 1.

**Table 1.** Mechanisms used by *V. cholerae* to resist host-specific colonization resistance.

Colonization resistance (CR)	*V. cholerae* resistance against CR	References
Reactive radical species	Produce catalases to metabolise ROS/RNS. Adjust biofilm morphology to resist ROS/RNS.	[15],[18],[20]
Bile	Taurocholate activates virulence. Use OMPs and RNS pumps for bile efflux. Use amino acid components to activate virulence.	[24],[34],[35]
Mucus	Activates virulence through T6SS. Use mucolytic enzymes and flagellum to lyse and propel into mucus respectively. Use mucin as an energy source.	[41],[45],[48]
Hypoxic conditions	Use alternate electron transport machinery. Hypoxia promotes virulence genes activation	[49]–[52]

### 2.1. Reactive radical species

In the gut, after invasion by a pathogen such as *V. cholerae*, the immune system modulates the production of reactive compounds, including reactive oxygen species (ROS) and reactive nitrogen species (RNS) that induce stress and hence antimicrobial effect in the gut limiting the survival of pathogens. It was illustrated that after *V. cholerae* infection, several proteins and enzymes are found to be elevated in the duodenal tissue, including dual oxidase 2 (DUOX2) and inducible nitric oxide synthase (iNOS), responsible for the production of ROS and RNS respectively [15]. ROS/RNS are known to be detrimental to bacterial cells by destroying their cellular and genetic materials, such as proteins, lipids, and DNA, and killing the bacteria. Counteractively, *V. cholerae* is known to resist ROS damaging effects due to its ability to produce specific catalases, including KatG and KatB enzymes, organic hydroperoxide resistance protein (OhrA), DNA binding proteins obtained from starved cells (DPS), chaperones, and peroxiredoxin [16],[17] that are crucially involved in the metabolism of ROS, nullifying its toxicities, and thereby providing ROS resistance. The ROS resistance enzymes and protein machinery are under tight regulation by *V. cholerae* virulence gene regulator, AphB, and quorum sensing systems that synergistically increase catalase production and biofilm formation [18]. Therefore, induction of ROS in the duodenal tissues benefits *V. cholerae* survival and colonization. At the same time, it remains a disadvantage to commensal microbes that cannot metabolize the ROS, limiting their growth and contributing to *V. cholerae* survival. Despite *V. cholerae* lacking the nitrate reductase enzyme, it can reduce nitrate at hypoxic and alkaline pH conditions [19]. It has been demonstrated that NorR, a nitric oxide-activated transcriptional regulator, upregulates the production of flavohaemoglobin, which is involved in RNS resistance in *V. cholerae* [20]. Furthermore, *V. cholerae* can resist reactive radical species toxicities in the gut by changing its morphology, switching between the rugose and smooth colony variation, and the change is reversible in response to the amount of reactive radical species [21],[22]. Moreover, once the pathogen establishes itself in the gut and produces cholerae toxin, eventually leading to diarrhoea, it will disrupt the microbiome and the ROS/RNS levels. Therefore, these findings demonstrated that *V. cholerae* has evolved several mechanisms to resist the toxicities of ROS/RNS species through the activity of specific catalases and by the pathogen's ability to form biofilm structures and switch between different forms of biofilm structures in response to the reactive species.

### 2.2. Bile acid/salt

Bile is composed of bile acids synthesized from cholesterol precursors in the liver and is conjugated with amino acids. It also contains cholesterol, immunoglobulin A (IgA), and phospholipids, which function as a digestive secretion to emulsify, solubilize, and aid the absorption of dietary fats [23]. In the human liver, the two predominant primary bile acids synthesized from cholesterol precursors include cholic acid and chenodeoxycholic acid; before they are transported out of the liver to the gallbladder, where they are stored before food ingestion, they are conjugated with one of two amino acids, commonly glycine, or taurine to form conjugated bile acids such as taurocholate or glycocholate [24]. The bile is secreted into the small intestine following food intake to mediate the digestion of fats and about 95% of bile acids are reabsorbed in the distal end of the ileum back to the liver while the remaining are metabolized by gut microbes into secondary bile metabolites [25],[26]. Distinctive from digestive functions, bile has antimicrobial activity by acting as a detergent aiding in the destruction of microbial membranes and their cellular homeostasis, thus posing a challenge to intestinal pathogens and restricting their colonization. For example, taurocholate was demonstrated to destabilize the matured biofilm structure of *V. cholerae* by changing the biofilm matrix and mediating the disintegration and detachment of the biofilm structure [27],[28].

However, several studies have demonstrated that *V. cholerae* is highly resistant to bile toxicities due to the pathogen's ability to restrict the influx of bile acids, using its OMPs, specifically the OmpU porin, to serve as efflux pumps for bile salts [29]–[31]. To achieve that, *V. cholerae* downregulate the expression of OmpT porin which function antagonistically to OmpU porin, and simultaneously upregulates the expression of OmpU porin [32],[33]. In addition to OMPs activity, an *in vitro* study, which was later supported by a rabbit model *in vivo*, it was indicated that *V. cholerae* resists intracellular bile salt toxicities by reducing the permeability of cell membrane to bile salt and upregulating the activity of *acrAB* gene that encodes for Resistance-Nodulation-Division (RND) efflux pumps, to contribute to pumping out the accumulated bile salts sensed in the cytoplasm [34]. In contrast to efflux pump activity, conjugated bile acids serve as signalling molecules for activating *V. cholerae* virulence gene expression. Taurocholate has been shown to promote the formation of intermolecular disulfide bonds in transmembrane transcription factor TcpP-a virulence gene regulator, mediating its dimerization and increasing its activity and subsequent *V. cholerae* colonization [35].

Furthermore, Xu et al. postulated that the reductase activity of a conserved oxidoreductase enzyme in bacterial periplasm, DsbA, is significantly inhibited in the presence of taurocholate. Still, its oxidase activity is increased, contributing to the formation of TcpP dimer, and hence improving TCP activity [36]. Interestingly, bile salts were shown to prevent the proteolysis of *V. cholerae* virulence regulon, ToxR, and promote the formation of its protein dimer and ToxRS complex [37]. The formation of ToxR protein dimers and the ToxRS complex facilitates ToxR activity [38]. Recently, it was also shown that the amino acid groups of the conjugated bile acid enhance the virulence mechanism of *V. cholerae* through the type VI secretion system (T6SS), thereby contributing to the killing of the gut microbiota [39]. This proves that the anti-biofilm activity of conjugated bile such as taurocholate is not entirely detrimental to *V. cholerae*, because the disassembly and dissociation of the biofilm structures due to taurocholate, reduced *V. cholerae* cell density, and on the other hand the autoinducer's activity in quorum sensing by the gut microbiota is delayed. Therefore, the taurocholate effect on *V. cholerae* virulence is more complex, from activating and enhancing the activity of *V. cholerae* major virulence regulon, ToxR, to aiding in exiting the biofilm structure and dissociation of the biofilm structure, both of which are advantageous to *V. cholerae* virulence.

The observations have shown that *V. cholerae* is highly tolerant of conjugated bile toxicity and that in the presence of conjugated bile acids and its amino acid component, the pathogen activates several virulence genes.

### 2.3. Mucus layer

The mucosal layers can be classified into two; the inner and outer mucus layers, where the inner mucus layer is highly defensive and attached to the gut epithelium forming a physical barrier that protects against bacterial colonization [40]. The outer mucus layer is heavily harboured and colonized by commensal microbes that metabolize microbiota-accessible carbohydrates (MACs) within the mucus and produce antibacterial metabolites, making the mucus even more challenging for invading pathogens to colonize. An individual diet is crucial in the maintenance of mucosal membrane integrity because a diet rich in processed foods and hence low in fibre and MACs, such as the western-style diet, leads to the slimming of the mucosal layer by mucus-degrading bacteria such as *Bacteroides* and *Akkermansia spp*., making the mucus vulnerable to bacterial colonization [41]. But a healthy diet maintains the composition of the gut commensal microbes, so the mucus integrity remains strong. *V. cholerae* can activate virulence mechanisms by sensing the presence of commensal microbes in the mucus and their antibacterial metabolites. This phenomenon is confirmed by several studies showing that the *V. cholerae* virulence factor, T6SS is activated in the presence of gut microbes and their metabolites *in vivo*; the T6SS serves as a molecular syringe to deliver toxins to the microbes and facilitates *V. cholerae* colonization [42]–[44]. In a separate study recently, to determine the significance and involvement of T6SS in *V. cholerae* colonization, T6SS mutants were designed and orally administered to mice, the results obtained proved that T6SS is important to *V. cholerae* successful colonization as the mutant strains show significantly lower intestinal colonization compared to wild-type *V. cholerae* with functional T6SS system [45]. Furthermore, *V. cholerae* can produce mucolytic enzymes, such as sialidases, peptidases, and sulphatases, to digest the mucus and, with the aid of its single flagellum, can propel itself into the thick mucosal layer to the intestinal epithelial surface to adhere [46],[47]. Also, a study by Wang et al. proved that the pathogen could utilize mucins as an energy source through the gluconeogenesis pathway during and after colonizing the gut, mucin is the major component of mucus [48]. These studies point out that the mucosal barrier is not a significant challenge for *V. cholerae* colonization, it evolved to digest the mucus, propel itself into it, utilize its component as an energy source, and the presence of protective gut commensals make the pathogen employ its weapon machinery T6SS to specifically kill the commensals to ease its colonization.

### 2.4. Hypoxic conditions

The low oxygen concentration in the gut is a factor in limiting the survival of several pathogens that cannot carry out anaerobic respiration. To start with, *V. cholerae* is a facultative anaerobe which gives it the advantage of surviving in oxygen-fluctuating environments by switching between aerobic respiration and fermentation depending on oxygen availability. Intriguingly, once the pathogen exits the oxygen-rich environment and enters the host upper intestines where oxygen concentration is low, a transcriptional analysis illustrated that the genes responsible for anaerobic metabolism are upregulated while those for aerobic metabolism are downregulated; this is a strategy to survive the oxygen tension [49]. Initially, under oxygen availability, *V. cholerae* favours aerobic metabolism to amplify in the epithelial crypt spaces by employing the pyruvate dehydrogenase (PDH) pathway instead of its anaerobic counterpart, pyruvate formate-lyase to produce acetyl-CoA from pyruvate for growth [50]. However, under oxygen stress, the pathogen has been shown to utilize several alternate electron acceptors, including nitrate, fumarate, and trimethylamine N-oxide (TMAO) [51]. Nitrate respiration gives vibrio a competitive advantage against gut commensals. Interestingly, CT production is enhanced by the anaerobic respiration of TMAO by the pathogen, as demonstrated in an infant mouse model by Lee et al. [52]. Several anaerobiosis processes modulate the activation of *V. cholerae* virulence factors. Liu et al. showed that *tcpP* expression is enhanced under anaerobic conditions, through the induction of AphB oligomerization by anaerobiosis [53]. Moreover, anaerobiosis promotes the formation of the ToxR-TcpP complex and is crucial in the induction of several *V. cholerae* virulence genes. These findings imply that the low oxygen concentration may be a competitive advantage to *V. cholerae* against some commensals since the pathogen is a facultative anaerobe, thus able to survive low oxygen concentration and the hypoxic nature of the distal gut leads to the activation of virulence genes.

## 3. Microbiota-specific colonization resistance mechanisms

**Figure 1.**
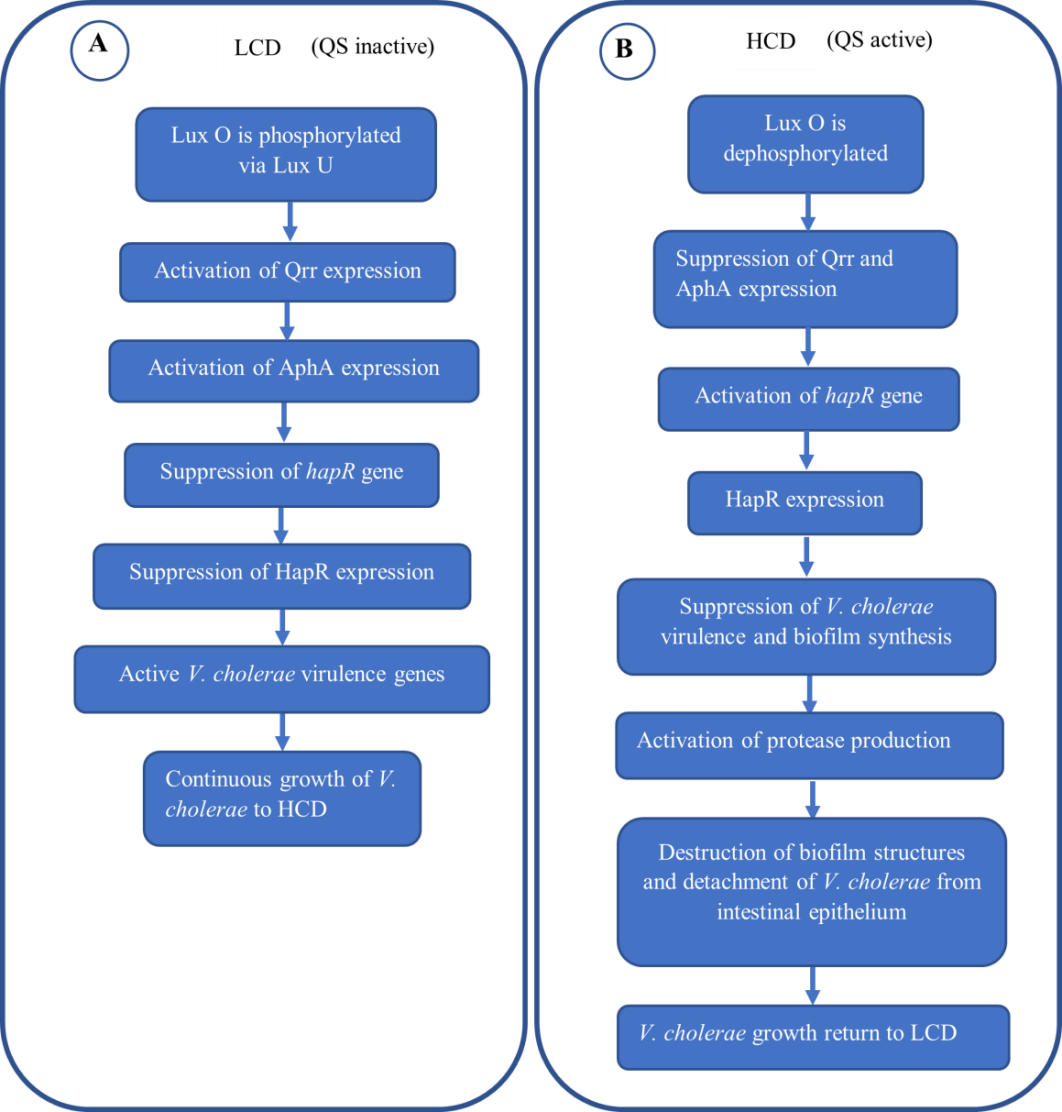
*V. cholerae* and quorum sensing at LCD and HCD. (A) In the early stages of infection, *V. cholerae* exits its biofilm structures as it enters the intestinal epithelium, at this point *V. cholerae* is at LCD. At LCD, Lux O is phosphorylated by Lux U, and the phosphorylated Lux O results in the activation of Qrr and AphA expressions. AphA expression suppresses the expression of the master QS regulator, HapR, thus QS will be inactive while *V. cholerae* virulence genes remain active, and the pathogen continues growing and colonizing the intestinal epithelium till it reaches HCD. (B) At HCD, the Lux O will be dephosphorylated and therefore both Qrr and AphA expressions will be suppressed while HapR expression will be activated, thereby activating the QS. When the QS is activated, *V. cholerae* virulence genes and biofilm synthesis will be suppressed and halted respectively. Subsequently, HapR activates the expression of proteases that aid in the detachment of *V. cholerae* from the intestinal epithelium. The detachment may aid in the further development of the disease due to subsequent dissemination of the pathogen in the gut and thus its growth return to LCD, which deactivates QS.

**Figure 2.**
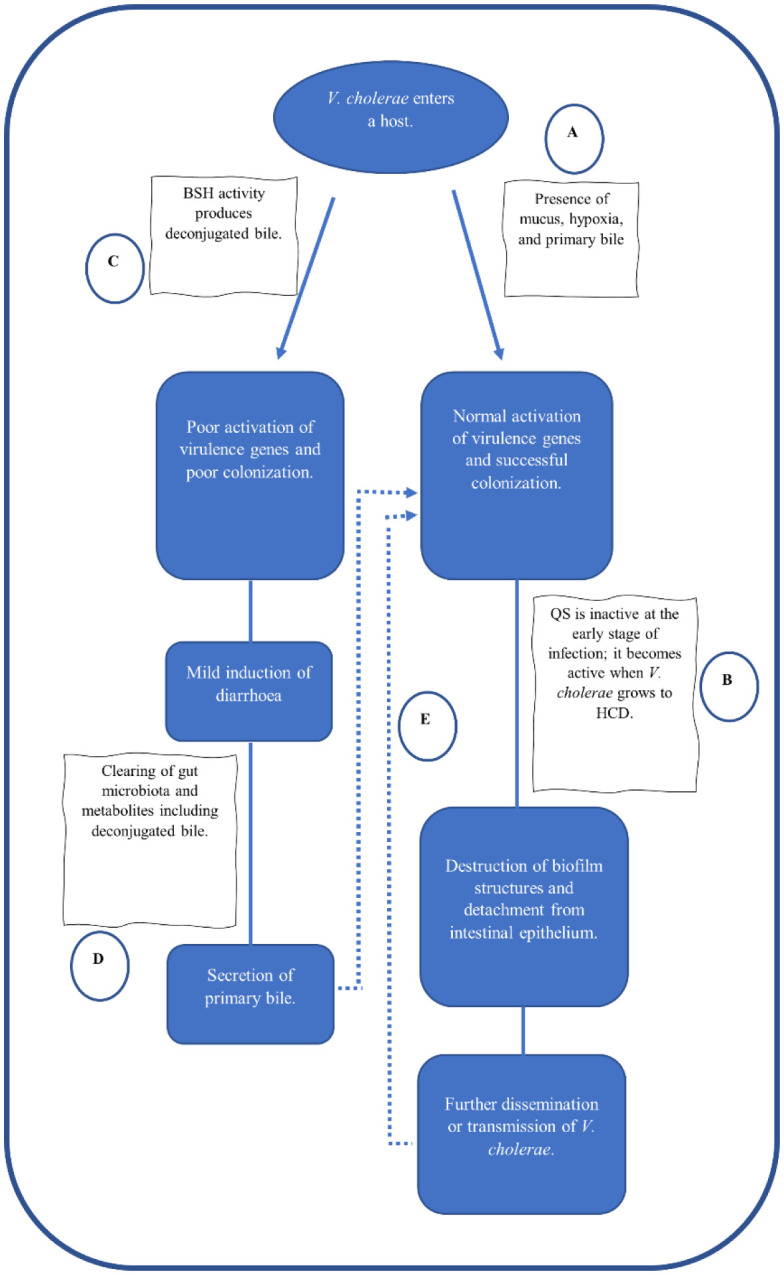
Relationship between *V. cholerae*, QS, and BSH activity. (A) When *V. cholerae* enters a host it senses the presence of mucus, hypoxia, and primary bile to activate virulence, that aid in the early colonization of the pathogen. (B) At this early stage of infection, QS is in active, because at early stage of infection, *V. cholerae* is in LCD. But as it continues to colonize and form biofilms structures in the intestinal epithelium, it reaches HCD, which then is sensed by QS, thereby activating it. When QS is activated, it results in the destruction of the biofilm structures and the detachment of *V. cholerae* from the intestinal epithelium. The detachment further disseminate *V. cholerae*, where is attach itself somewhere or it exit through diarrhoea, further transmitting the infection. (C) However, in the presence of commensals that have BSH activity, the deconjugated bile result in poor activation of virulence and thus poor colonization and induction of mild diarrhoea. (D)The mild diarrhoea result in clearing of the gut microbiota and metabolites, thereby the secretion of primary bile is restored. (E) The subsequent secretion of primacy bile due to mild diarrhoea and the further dissemination of *V. cholerae* due to QS will eventually result into another cycle of infection, by activating of virulence by primary bile and *V. cholerae* going back to LCD thereby inactivating QS, respectively.

Some colonization resistance mechanisms are derived explicitly from the gut microbiota (summarized in Table 2, Figure 1 and 2). These mechanisms are even more challenging to *V. cholerae* than host-specific mechanisms due to their specificity, highlighting the significant role of microbiota in protecting against invading pathogens.

**Table 2.** Mechanisms used by *V. cholerae* to resist gut microbiota-specific colonization resistance.

Colonization resistance (CR)	*V. cholerae* resistance against CR	References
Quorum sensing (QS)	QS active only at HCD. Use CAI-1 to induce diarrhoea. QS is active at HCD and aids in further disseminating and transmitting *V. cholerae*.	[1],[69]–[72]
Nutrient competition	Able to utilize mucin as an energy source. Able to obtain Fe from RBC.	[83],[88],[90]
Bacteriocins	Utilize OMPs and RND pumps to neutralize the bacteriocins effect.	[99],[100]
SCFA	Consumes acetate. Kills SCFA-producing bacteria. Use NhaP1 anti-porter to maintain pH. Induce diarrhoea to flush out commensals.	[107],[111]
BSH	Flush out commensals, thus resulting in the restoration of virulence-stimulating primary bile salts.	[114],[118]

### 3.1. Quorum sensing (QS)

QS refers to intra- and inter-bacterial species cell-cell communications through diffusible chemical signals known as autoinducers to monitor the population density of a microbial community [54],[55]. These small molecular weight chemical molecules are produced by many gram-positive and gram-negative types of bacteria to control several physiological functions and limit the concentration of microbial species. In general, both types of bacteria are known to produce (2S, 4S)-2-methyl-2,3,3,4-tetrahydroxytetrahydrofusan borate (AI-2), which is classified as a furanyl dibasic molecule. In contrast, acyl-homoserine lactone (AHL) is known to be distinctive to gram-negative bacteria only, and small-molecule polypeptides are specifically produced by gram-positive bacteria [56]. Intriguingly, *V. cholerae* virulence can be modulated by autoinducers which can affect the activity of the complex and well-coordinating virulence regulatory cascade of *V. cholerae*, thereby affecting the pathogen's colonization potential [57]. The commensal microbes of the gut are known to produce the cross-species autoinducer, AI-2, while cholera autoinducer (CAI-1) was reported to be produced only by *Vibrio species* naturally; both autoinducers have been demonstrated to modulate *V. cholerae* virulence by signalling its gene regulation [58],[59].

Moreover, *V. cholerae* is illustrated recently to produce a third autoinducer molecule known as 3,5-dimethylpyrazin-2-ol (DPO) [58]. *Blautia obeum*, a gut commensal microbiota, has been shown to significantly reduce *V. cholerae* colonization by upregulating its production of AI-2 after sensing the presence of *V. cholerae* in the gut of germ-free mice [60]. The AI-2 modulates virulence in a LuxS-dependent manner independent of LuxP; several gut microbes have been reported to harbour LuxS homologs in their genome. Deletion of the *luxP* gene of *V. cholerae* was reported by Hsiao et al. to be ineffective in resisting the effect of AI-2 from *B. obeum* [1]. The role of AI-2 was determined in patients recovering from cholerae infection, the existing gut commensal microbiotas that first colonize the gut after *V. cholerae* infection are shown to produce AI-2 crucial in inhibiting *V. cholerae* further virulence [60],[61]. In another study aimed at studying the role of CAI-1, *E. coli* was engineered to express and produce CAI-1 in an infant mouse model, the CAI-1 demonstrated an 80% reduction in cholera toxin B (CTB) binding to the epithelial surface, thereby preventing virulence and colonization of *V. cholerae* [62]. This shows that the CAI-1 may be involved in the *V. cholerae* intra-specie competition.

However, the QS is only effective when the *V. cholerae* is at high cell density (HCD), at a later stage of the infection. But at the early stage of infection, when the *V. cholerae* is in low cell density (LCD), the concentration of the autoinducers is low and not enough to suppress the virulence genes, as a result, the virulence genes remain active, including the biofilm biosynthetic gene [63],[64]. When *V. cholerae* enters the host, it exists in a biofilm structure which is crucial upon entry by protecting stomach pH [65],[66], but once it reaches the intestine, the biofilm structure is exited, and individual *V. cholerae* cells disperse into the intestine, and the cell density is extremely low. At this LCD, LuxO is phosphorylated by the *V. cholerae* QS regulatory system via LuxU, which acts as a phosphotransferase in the presence of a kinase (QS regulatory components) [67],[68]. The phosphorylated LuxO subsequently activates the expression of quorum regulatory RNAs (Qrrs), and the Qrrs subsequently induce the activation of one of the four *V. cholerae* virulence transcription activator, AphA, and suppress the activation of the *hapR* gene, which encodes for the master QS regulator, HapR [69]–[71]. This implies that the QS is inactive at the early stage of infection, and the virulence genes are active, allowing *V. cholerae* colonization and expression of other virulence factors. But when the individual *V. cholerae* cells grow to HCD, the level of autoinducers rises and the phosphorylated LuxO will be dephosphorylated. This eventually results in the suppression of Qrrs and AphA expressions and the activation of the *hapR* gene that results in the expression of HapR that suppresses virulence gene expression including biofilm biosynthesis and induce the production of proteases that lead to the detachment of the *V. cholerae* from the epithelium of the intestines [72],[73]. However, the detachment is not completely destructive to the *V. cholerae*, as it prepares the pathogen for exiting the host or reattaching itself somewhere in the intestine; thus, it ends up aiding in the transmission or dissemination of *V. cholerae* respectively [74]. Thus, QS is effective at HCD and may benefit the pathogen in transmission and further dissemination, while the QS remains inactive at LCD, as summarized schematically in Figure 1. Furthermore, the QS of *V. cholerae* is shown to function by affecting the physiological function of other enteric microbes to their advantage. Surprisingly, Gorelik et al. reported that the CAI-1 from *V. cholerae* affects *E. coli* (EPEC) E2348/69, by inducing the expression of the *E. coli* type-3 secretion system (T3SS), which is known to induce diarrhoea, showing that CAI-1 can affect non-*Vibrio species* [75]. *V. cholerae* inducing *E. coli* to cause diarrhoea is a strategy to enhance the clearance of gut microbiota, thus contributing to the colonization of *V. cholerae*.

### 3.2. Nutrient competition

When an invading pathogen enters the gut, it has to compete with the gut commensals for nutrients normally limited in the habitat. The nutrient competition can be in terms of macronutrients such as amino acids and carbohydrates for energy sources, micronutrients such as vitamins, and trace metals crucial to several enzymes. This competition can limit the colonization potential of several enteric pathogens. Nutrient competition is more competitive when bacterial strains require similar metabolic nutrients, as it exists between strains of the same species [76],[77]. The gut mucus is multifunctional because apart from its primary function as a physical barrier to protect the epithelial cells, it serves as a reserve of carbohydrates that several gut commensals and even invading pathogens such as *V. cholerae* use as an energy source. The commensal microbiotas are in homeostasis with the concentration of nutrients available in the gut mucosa. Still, in the presence of invaders, the mucosal integrity may be lowered due to the degradation of the mucus by the enteropathogens that utilize mucus for energy [41],[50]. A variety of carbohydrates are identified in mucus, including fucose, sialic acid, N-acetylgalactosamine (GalNAc), and N-acetylglucosamine (GlcNAc) [78]. Several reports demonstrated that *V. cholerae* contains genes responsible for GlcNAc and sialic acid metabolism. The same genes are found in many gut commensals, including *Akkermansia muciniphila*, *Bacteroides*, *Lactobacillus,* and *Bifidobacterium* as reported in different studies [50],[79]–[81]. Despite the competition from these commensals, *V. cholerae* can utilize the metabolites, mediating its pathogenicity.

Consequently, when *V. cholerae* genes involved in the utilization of sialic acid and GlcNAc were inactivated, the gut colonization potential of the pathogen was reported to significantly decrease [82]. Reddi et al. reported that the two macromolecules are carbon sources and promote *V. cholerae* motility through the thick mucosal layer [83]. Advantageous to enteric pathogens, including *V. cholerae*, it was shown that the presence of glycans- and sialic acid-utilizing commensals may result in the susceptibility of enteric infection due to overall enhanced degradation of the mucosal nutrients, thereby weakening the mucus thickness and integrity [41],[84].

*V. cholerae* is demonstrated to alternatively use L-ascorbate (Vitamin C), a micronutrient, as an energy source, and it has to compete with the commensal microbiota for it. To determine the role of L-ascorbate in *V. cholerae*, Rosenberger et al., showed that *V. cholerae* with defective L-ascorbate utilization machinery is unable to compete *in vitro* when grown in an M9 minimal media with supplementation of casamino acids and intestinal mucus, and they conclude that the fermentation of the metabolite may be significant in an *in vivo* phenotype [85]. Some commensal anaerobes can produce several essential vitamins for the human body, including vitamin K and the water-soluble vitamin B12 [86]. Many microbes, including *V. cholerae*, cannot synthesize B12 de novo, and thereby have to obtain it competitively through transport mechanisms [87]. However, knocking out of genes involved in the uptake of corrinoid, an intermediate in the carbamide-dependent methionine synthase, could not affect the colonization potential of *V. cholerae* in suckling mice [88] indicating that the corrinoid is not critical to *V. cholerae* colonization.

The amount of trace metals in the human body is tightly regulated; therefore, both the commensal and the invading pathogens must compete for them. These metals, including Fe, Zn, and Cu, are critical to the activity of several critical enzymes, serving as a cofactor or a prosthetic group to them, by which the activity of the enzyme is null without it [89]. In *V. cholerae*, Zn is highly significant in its growth and colonization. Sheng et al. illustrated that when the genes responsible for utilizing Zn and regulating the amount of Zn are deleted, the pathogen shows significant growth and colonization defects in germ-free mice [90]. Moreover, they further demonstrated that such a defect is severe in the presence of gut commensals. Intriguingly, *V. cholerae* does not have to compete severely for Fe with the commensals. Because most of the Fe in the body is not free but is a component of heme, and the CT of *V. cholerae* can force the Fe out of the heme by inducing mechanisms that result in the congestion of the blood capillaries in the ileum carrying red blood cells, thereby releasing free heme and Fe, and thus acquiring the Fe for its utilization [91],[92].

Therefore, *V. cholerae* gut colonization is not significantly affected by nutrient competition by the gut commensals, especially for macromolecules. However, trace metals such as Zn and Fe are crucial for *V. cholerae* growth and colonization; while it can obtain Fe through CT activity, it must compete for the limited amount of Zn for its survival.

### 3.3. Bacteriocins

Bacteriocins are a kind of short peptide molecules with antibacterial activity produced by gram-positive and gram-negative bacteria that affect the growth and colonization of other bacterial species [93]–[95]. They act by creating pores in the cell membrane and also interfere with the genetic material of the bacteria through DNA and RNA metabolism, thereby killing the cells [96]. Bacteriocins have a wide range of activity; the bacteriocins from gram-positive bacteria can effectively inhibit gram-negative bacteria. This is consistent with enormous research on the effect of bacteriocins on *V. cholerae* growth. More than 3 decades ago, an *in vitro* study using a bacteriocin produced by a gram-positive bacterium, *Streptococcus lactis* 11451, was shown to provide an inhibitory effect on the growth of *V. cholerae* [97]. Casein A, another bacteriocin from gram-positive bacteria, *Lactobacillus casei* OGM12, isolated from selected fermented food, was shown to block the growth of *V. cholerae in vitro* [98].

Moreover, a bacteriocin from *Pediococcus acidilacticii* QC38, also a gram-positive bacterium, was shown to have an antagonistic effect on *V. cholerae*. The study was carried out *in vitro*, and the culture supernatant of the bacterium used was isolated from a food product containing the bacterium [99]. These reports indicate that bacteriocins from gram-positive probiotics can effectively inhibit *V. cholerae* growth *in vitro*. However, the mechanism by which these bacteriocins from gram-positive bacteria act on *V. cholerae*, a gram-negative bacterium, is not well understood because the presence of outer membrane protein (OMP) should mask the bacteriocin from binding to *V. cholerae*. The effect of bacteriocins from gram-negative bacteria on *V. cholerae* has not been an active research area. But several studies have been conducted to show the resistance mechanism of *V. cholerae* to bacteriocins, as mentioned earlier. OMPs and vesicles are crucial in the resistance against these antibacterial peptides. In the presence of bacteriocins, the content of the vesicular secretion by the *V. cholerae* remains unaffected, but the structure of the vesicles will be altered concerning the protein composition, including Bap1 protein and two important components of OMPs, OmpV, and OmpW. The Bap1 protein was demonstrated to be involved in scavenging the antibacterial peptides as a strategy to resist their toxicity. The Bap1 protein was shown to combine with another outer membrane protein, OmpT, serving as a ligand in the scavenging [100]. Also, in a recent study, another class of OMP, OmpA, was demonstrated to interact with SipA, a form of periplasmic space protein, and together they provide a mechanism to resist antibacterial peptides. Once the bacteriocin enters the periplasmic space, the SipA protein may bind to it and then present it to OmpA to export it out of the periplasmic space, thereby preventing the bacteriocin effect [101].

Interestingly, the RND efflux pumps are significant in providing resistance to *V. cholerae* against the destruction by antimicrobial peptides. This was stipulated by Bina et al., who studied the RND mutants of *V. cholerae* and discovered that the mutants are highly irresistible to antimicrobial peptides *in vitro*. A further *in vivo* study indicated that the mutants show a colonization defect in infant mouse studies [102]. Therefore, *V. cholerae* has evolved mechanisms to resist the destructive effect of bacteriocins against its survival and colonization. There are limited studies on the impact of bacteriocins on *V. cholerae* colonization; further studies should be carried out to detail the effect of bacteriocins from gram-negative bacteria on *V. cholerae* and compare the effect between the two grams.

### 3.4. Short-chain fatty acids

The fermentation of indigestible carbohydrates and resistant starch from dietary fibres by gut microbiota results in the production of SCFA, mainly composed of acetate, butyrate, and propionate, while other forms of SCFA make up about 5–10% of the gut SCFA pools [103]. The SCFA are more than mere digestion metabolites; they play a key role in maintaining gut immunity through immune homeostasis, maintaining mucosal integrity and anti-inflammatory effects, and serving as an energy substrate for the gut epithelial cells [104]. They act as bioactive molecules that have antibacterial activity by affecting bacterial metabolic function, disrupting its intracellular pH, inhibiting growth, and providing colonization resistance against invading pathogens. Their antibacterial activity is at most at a lower pH because SCFA exists in nonionized forms at that pH, and therefore they can quickly enter bacterial cells through diffusion. In the bacterial cytoplasm, they dissociate into cations and anions, which subsequently build-up and lower the bacteria's intracellular pH [105],[106]. The gut microbiota is also known to modulate the number of ROS/RNS species through SCFA metabolism. Metabolism of SCFA, such as butyrate, is known to reduce the amount of ROS. The reduction of ROS, on the other hand, can be of advantage to pathogens [107].

Counteractively, *V. cholerae* has evolved mechanisms to resist the toxicities of the SCFA. In the presence of acetate, *V. cholerae* upregulates the activity of acetyl-CoA synthase-1, which is central in the conversion of the acetate in the cytosol of the pathogen, thus depleting the intestinal acetate concentration. This depletion of intestinal acetate by *V. cholerae* may affect the host insulin signalling pathway, blocking its signal transduction, and leading to lipids accumulation, affecting the overall individual's health [108]. Moreover, once *V. cholerae* established itself in the gut, it can also decrease the amount of SCFA by decreasing the amount of SCFA-producing gut commensals such as *Bifidobacterium species*. This was reported in a clinical surveillance sample, demonstrating the decrease in abundance of *Bifidobacterium species* during the infection, and the level is restored to normal as the treatment of the infection progresses [109]. You et al. reported that when mice were treated with antibiotics, such as clindamycin which clears the abundance of *Bacteroides species*, a decrease in the level of SCFA was observed and an enhanced colonization potential by *V. cholerae* [110]. This report has shown the role of Bacteroides species in producing SCFA as bioactive molecules in providing anti-colonization mechanisms.

Furthermore, *V. cholerae* grew even under low pH conditions; it utilizes the antiporter of K^+^/(Na^+^/H^+^), NhaP1, to maintain its internal pH. The antiporter function by taking in H^+^ and taking out K^+^ or Na^+^, the constant uptake of H^+^ from the cytoplasm decreases the pH, while the H^+^ taken in by *V. cholerae* is depleted through respiratory pathways [111]. Moreover, the cholera toxin produced by *V. cholerae* causes electrolyte imbalance, resulting in diarrhoea and providing the pathogen with a suitable environment for its growth and colonization. This implies that the colonization potentials of *V. cholerae* can be enhanced through the depletion of SCFA-producing gut commensals through diarrhoea, and *V. cholerae* employs the strategy to resist colonization resistance concerning SCFA toxicities.

### 3.5. Bile salt hydrolase (BSH)

As mentioned earlier in this review, primary bile salts secreted by the liver for digestive purposes are conjugated with amino acids such as taurine or glycine, and the conjugated form has shown antimicrobial activity against microbes. This antimicrobial activity of bile is a human-derived colonization resistance mechanism that *V. cholerae* has evolved a mechanism to resist its effect. *V. cholerae* utilizes taurocholate, a primary bile salt, to activate virulence in a *tcp*-dependent manner. Intriguingly, the gut commensals that inhabit the small intestines also devise mechanisms to resist the bile acid toxicities by destroying the detergent-like nature of the bile [112]. This is achieved by using a bile salt hydrolase (BSH) enzyme that deconjugates the primary bile acids by catalyzing the removal of the conjugated amino acids from the bile acids. Several commensals in the gut are shown to contain the *bsh* genes as a survival mechanism against bile in the intestine [113]. A recent study by Song et al. illustrated that the *bsh* phylotypes from different commensals are not the same and have different substrate-dependent deconjugating activity [114]. The same study demonstrated that the BSH enzyme in *B. obeum* showed higher deconjugating activity against taurocholate, taurodeoxycholate, glycocholate, and glycodeoxycholate. The gut microbial deconjugation of bile acids plays a significant role in their bile tolerance and circulation, thus providing colonization resistance against *V. cholerae*. Deconjugation of taurocholate by *B. obeum* was reported in a recent study to affect the colonization potential of *V. cholerae*, due to the poor expression of virulence genes, and this can be explained as taurocholate is critically involved in the activation of virulence genes, and hence aid in colonization [115]–[117]. These studies concluded that the commensal microbiota, *B. obeum*, plays a crucial role in driving *V. cholerae* colonization resistance via deconjugation of taurocholate, and the BSH activity affects the pathogenesis of the *V. cholerae* the most at the early stage of infection. The significance of *B. obeum* amongst other commensals with the *bsh* activity was demonstrated in a mouse model; the removal of *B. obeum* was shown to significantly raise the level of taurocholate in the mouse's distal small intestine [57]. Moreover, a healthy gut microbiome plays a vital role in overall BSH activity; this was shown in an *in vitro* study, where a gut microbiome was obtained from faecal samples of healthy and non-healthy Bangladeshi individuals. The microbiome of healthy subjects displays a high abundance of *bsh* genes and inhibits *V. cholerae* colonization. In contrast, the dysbiotic microbiome from non-healthy subjects shows a decreased abundance of *bsh* genes and a microbiome susceptible to *V. cholerae* infection [115]. It is also illustrated that the gut microbiome's intestinal balance of different forms of bile acids concerning the constitutive BSH activity is essential in determining *V. cholerae* expression of TCP at an early stage of colonization. Thus, King et al. showed that the early *tcp* gene expression by *V. cholerae* in response to the constitutive BSH activity from gut commensal may be correlated with the determination of infection progression variations, which may result in either symptomatic or asymptomatic infection due to full or partial colonization of the pathogen respectively [118].

It is worthy of note that once *V. cholerae* establishes itself in the gut and begins inducing diarrhoea, subsequently, diarrhoea flushes away the gut commensal microbiome and bile metabolites, such that subsequent secretion of bile will be the conjugated primary bile acids such as taurocholate that mediates *V. cholerae* virulence [57]. This explains why the effect of BSH is more important at the beginning of infection, but once *V. cholerae* is established in the gut, the BSH activity is diminished. On the contrary, the BSH effect on *V. cholerae* colonization is less clear because other contradictory reports indicate that the deconjugated bile acids are also involved in the activation of virulence through ToxR and ToxS synergistically enhancing the activity of TcpP in binding to *toxT* promoter [119],[120]. Also, the deconjugated bile salts are argued to stimulate *V. cholerae* to activate some genes involved in forming a biofilm that provides resistance to the bile salt. Hung et al. reported that when *V. cholerae* is not in biofilm form in response to the toxicities of deconjugated bile salts, it activates the transcriptional activator of *vps* and *vpsR* genes and hence the formation of biofilm structure against bile toxicities [120]. However, more reports are indicating the poor activation of virulence in the presence of deconjugated bile [57], [112]–[118], therefore, future studies are required to fully understand the effect of BSH activity on *V. cholerae*.

Conclusively, the BSH activity significantly inhibits *V. cholerae* colonization at the early stage of infection in the presence of healthy gut microbiota, but during the later stage of the infection, when symptoms begin, the effect subsides. And *V. cholerae* also devise mechanisms to resist the toxicities of the deconjugated bile salts by upregulating its efflux pumps and enhancing biofilm formation.

## 4. Gene expression and regulation

*V. cholerae* inhabits two entirely different environments, the aquatic environment, and the host gut, that require different survival mechanisms to grow and adapt to that environment, and therefore while it transits from one environment to another, it must regulate its gene expression to adapt to the new environment. When *V. cholerae* transit from environmental reservoirs to the human host, it downregulates the expression of some specific genes involved in survival in the external environment and upregulates the genes that aid in its adaptation in the human gut. The pathogen tightly regulated this gene regulation and expression, using several host and microbiota-specific signals to modulate the gene expression. The gene expression will favour the expression of several virulence genes mediating the pathogen's growth and colonisation during the infection.

The pathogen enters the human host in biofilm form, and the biofilm structure is crucial at the early entry stage as a mechanism to resist the inhospitable stomach environment [121]. *V. cholerae* produces a Type IV mannose-sensitive hemagglutinin (MSHA), a structure that is involved with the biofilm formation while the pathogen is in the external environment; however, host-specific secretory antimicrobial; IgA was reported to be non-specifically recognize and bind to the pathogen through the recognition of the MSHA structure on the biofilm [122]. This may pose a challenge to *V. cholerae* survival and hence colonization because this will result in the clearance of the pathogen in bulk flow, as bacteria that are captured by the secretory IgA can be entrapped in the outer mucosal layer and hence susceptible to the bulk flow clearance to remove them from the gut [123],[124]. To avoid this obstacle, it was illustrated that *V. cholerae* represses the expression of genes involved in the biogenesis of MSHA, through a crucial *V. cholerae* virulence modulator, ToxT [125],[126]. Furthermore, a separate study demonstrated that among the genes of *V. cholerae* that are repressed during infection are the genes that encode for an ion exchange transporter, the H^+^/Cl^−^ transporter [127]. This transporter is encoded by the *clcA* gene, which plays a key role at an acidic pH such as during the early stage of entry in the stomach due to the acidic nature of the environment, but in the intestines where the pH is higher, these transporters can inhibit colonization efficiency.

Conversely, the genes that play a significant role in the colonization and virulence of *V. cholerae* are induced and upregulated in a tightly regulated manner. In *in vitro* studies, to understand the upregulated genes during the early stage of *V. cholerae* colonization in the gut, an artificial condition needs to be established which is entirely distinctive from the *in vivo* conditions; this is because the virulence genes are often expressed sufficiently and accurately *in vivo* conditions only [128],[129]. However, with the emergence of a recombinant-based in vivo expression technology (RIVET), these studies can be observed *in vivo*, and RIVET was used to study the expression of *V. cholerae* virulence based on time and at an individual *V. cholerae* cell level [130]. This study revealed that the *tcpA* gene that encodes TCP is upregulated, and its induction proceeds biphasically during the infection in separate temporally and spatially exclusive events. They stipulated that ToxT alone is indispensable in activating *V. cholerae* virulence *in vivo*, as opposed to *in vitro* conditions where ToxR and TcpP are equally needed to activate virulence genes. Mandlik et al. later suggested that the *V. cholerae* virulence activation *in vivo* is highly complex after they employed RNA-seq analysis to identify the elevated genes in animal models at the early stage of infection and observed that some genes that are not involved with virulence are also elevated at the stage [131]. Using the same RIVET technology, the genes upregulated later during *V. cholerae* infection when the pathogen has established itself in the gut are studied [132],[133]. The results showed that the genes involved in the pathogen's metabolism such as gluconeogenesis or citrate fermentation and genes encoding for anti-colonization such as MSHA or those involved in the survival outside the host are expressed. This explains that the pathogen prepares to exit the host to the external environment. Interestingly, DiRita et al. reported that ToxT is central to the positive and negative gene regulation of the colonization and anti-colonization factors of *V. cholerae*, explaining the pathogen's efficiency in combining signals from both the host and gut microbiota to simultaneously upregulate or downregulate genes [134].

## 5. Gut microbiota-derived cholera susceptibility

A healthy gut microbiota decreases *V. cholerae* susceptibility through various colonization resistance mechanisms, as mentioned earlier in this review. The gut microbiota plays a key role in *V. cholerae* infection in animal models, including mice, rabbits, and guinea pigs. A simple pre-treatment with an antibiotic is enough to eliminate and disrupt the commensal gut microbiota which results in enhanced *V. cholerae* colonization [135]. The gut of adult mice is resistant to *V. cholerae* infection, and to allow the colonization of *V. cholerae*, the gut microbiota must be depleted with antibiotics [136]–[138]. Some commensal microbiota species have been demonstrated to be crucial in the *V. cholerae* colonization resistance. In germ-free mice, prior colonization with *B. obeum* is demonstrated to inhibit *V. cholerae* colonization through two major mechanisms; by deconjugating taurocholate using its BSH enzyme and by the production of AI-2 which inhibits *V. cholerae* biofilm formation [115],[139]. Also, another gut commensal microbiota, *Bacteroides vulgatus* was illustrated to significantly reduce *V. cholerae* colonization and proliferation through the production of SCFA via the metabolism of host glycans in germ-free adult mice [110]. Therefore, the depletion of these commensals will lead to cholera susceptibility.

Conversely, some gut microbiotas increased the susceptibility of *V. cholerae* infection, such as *Paracoccus aminovorans*. Barrasso et al. reported that *P. aminovorans* is involved in the enhancement of *V. cholerae* biofilm formation, through the formation of dual-species biofilm structures between *V. cholerae* and *P. aminovorans* [121]. Surprisingly, during *V. cholerae* infection, the diarrheal activity is shown to deplete the gut microbiota, but *P. aminovorans* are found in significant abundance during the onset of the infection [140]–[142]. This shows that the association between the two species is significant, even though rather unusual. Furthermore, in an *in vitro* study, it was illustrated that the *P. aminovorans* increase and enhance *V. cholerae* biofilm formation only in nutrient-rich environments, while in limited nutrient environments, no significant increase is observed [143]. They suggested that in a nutrient-rich medium, *P. aminovorans* may produce a key metabolite that serves as a substrate for the growth of *V. cholerae*. This is a distinctive stipulation from the Barrasso study, which deduced the interaction based on the formation of dual biofilm between the two bacteria, showing that their relationship may be more complex. Another possible explanation is that *P. aminovorans* may facilitate the growth or enhance the formation of *V. cholerae* biofilm by reducing the level of reactive oxygen species. Further studies are needed to further understand this relationship.

However, several studies have been conducted to further pinpoint which microbiota is likely to result in the susceptibility of cholerae infection. The results are conflicting because some commensal microbiota was reported to be abundant in a healthy gut and also during *V. cholerae* infection. This includes several Enterobacteriaceae taxa and even *Ruminococcus* and *Blautia* which are known to provide resistance to *V. cholerae* infection [144],[145]. This is discordant with other observations demonstrating the clearance of *B. obeum, B. buccalis, B. ovatus and B. uniformis* during infection through diarrheal activity [141]. *Prevotella species* were found to be in high abundance in healthy individuals, and using machine learning studies, it is reported to provide resistance to *V. cholerae* infection [146],[147].

In summary, the gut microbiota is crucial in determining individual susceptibility to enteropathogens, including *V. cholerae* infection, directly or indirectly through the modulation of host immune systems. Diet plays an essential role in maintaining healthy gut commensals; therefore, a diet that decreases the abundance of gut commensals or, in case of malnutrition, may drive an individual towards susceptibility to cholera infection. Low and middle-income countries where poverty is high and hence malnutrition explain reasons that contribute to the high rates of cholera infection.

## 6. Future perspectives

An in-depth understanding of how *V. cholerae* overcomes the colonization resistance mechanism may pave a new way in therapeutics and diagnosis of cholera. Several research has been conducted *in vitro* and *in vivo* to explain the mechanisms of colonization of *V. cholerae* using different molecular and bioinformatic tools; however, research needs to be done to join the different studies. The studies focusing on the interaction between the gut microbiome and *V. cholerae* have revealed several understandings of the evolution of *V. cholerae* to curtail the antagonistic effort by the microbiota in preventing its colonization; however, some of the reports are contradicting or not fully understood [1]. For example, some reports indicate that deconjugated bile salt reduces *V. cholerae* virulence due to poor activation of the pathogen's virulence factors, while other reports suggest that the deconjugated bile is involved in the activation of genes that promote virulence. Therefore, studies that involve multiple or combined approaches may be required to identify the specific role of deconjugated bile in *V. cholerae* virulence.

Further research is needed to support the phenomenon and other conflicting results. Using multi-omic approaches, the impact of *V. cholerae* on the gut and the host may be further illustrated and can be beneficial in vaccine development and therapies. Moreover, combinatorial approach studies should be carried out to study the synergistic effect of two or more colonization resistance mechanisms in providing resistance against *V. cholerae* infection.

Furthermore, concerning animal models, the gut of adult mice model frequently used in previous studies is resistant to *V. cholerae* infection, and as such prior treatment with antibiotics is necessary. This treatment may affect several outcomes concerning the interaction of the pathogen and a healthy gut microbiome, making it difficult to mimic normal gut conditions [136],[148]–[150]. In rabbit models, ligated ileal loops are needed because oral infections are impossible to achieve [151],[152]. Therefore, newer models are required to fully understand the interaction. As such, zebrafish [153],[154] and fly models [155],[156] are recently introduced but pose challenges such as the inability to define gut microbiota for a sustained period and CT-independent toxicities in zebrafish and drosophila, respectively.

## 7. Conclusions

The human gut is exposed to tons of microbes every day, and as a result, the gut and its commensals team up and stay alert to protect the gut from invading pathogens such as *V. cholerae* using different colonization resistance mechanisms. The colonization resistance mechanisms serve as lines of defence, challenging the growth and colonization of invading pathogens, thereby limiting their existence. Despite these layers of defence, *V. cholerae* can grow, colonize, and cause cholera infection by neutralizing the colonization resistance mechanisms, even though the colonization efficiency and severity of the cholera infection may differ depending on how challenging the colonization was.

*V. cholerae* has evolved mechanisms to survive in the human host, using the colonization resistance as signals to activate virulence and aid in colonization. *V*. *cholerae* is a facultative anaerobe that allows it to grow in both low and high oxygen concentrations and contains alternate electron transport machinery, and it is single-flagellated and serves as a propeller to the pathogen. These characteristic natures of the pathogen are enough to break several layers of host-specific colonization resistance mechanisms. *V. cholerae* is shown to be resistant to ROS/RNS by the action of catalases and alternate electron transport machinery, low oxygen concentration by its facultative anaerobic nature, conjugated bile serving as a virulence gene activator and the OMP prevent bile influx, and the thick mucosal layers of the gut are degraded by its mucolytic enzymes. At the same time, the single flagellum propels it into the mucus to the gut epithelial cells. However, the gut microbiota-specific colonization resistance mechanisms require a more sophisticated process to resist, and *V. cholerae* evolved mechanisms to overcome them. The bacterial QS does not prevent colonization entirely because it is only active at HCD, and at the early stage of infection, the *V. cholerae* is in LCD. *V. cholerae* utilize its OMPs and RND to neutralize the effect of bacteriocins, while the effect of SCFA and low pH are antagonized by using a specialized antiporter that increases the pH by scavenging H^+^. The activity of the BSH enzyme is only effective at the early stage of infection, and the deconjugated bile salts produced are arguably shown to activate virulence. All of the above demonstrates that *V. cholerae* has evolved mechanisms to survive in the host, even though some mechanisms are not clearly illustrated, and further studies are required to fully understand them. Moreover, the pathogen regulates its gene expression majorly through ToxT, to switch between genes required for survival in the two distinctive ecological niches it inhabits. This is because the genes upregulated outside the host may not be required in the human host and can serve as an anti-colonization factor; thus, they are downregulated.

Conclusively, the gut microbiome is central in determining cholera susceptibility; dysbiotic microbiota cannot provide sufficient colonization resistance mechanisms, and the gut will be vulnerable to *V. cholerae* colonization. A healthy gut microbiome may be challenging to *V. cholera* colonization such that even if the colonization is successful, it will be mild, and hence the individual will be less susceptible to *V. cholerae* infection.
